# Human amniotic fluid derived extracellular vesicles attenuate T cell immune response

**DOI:** 10.3389/fimmu.2022.977809

**Published:** 2022-11-28

**Authors:** Tania del Rivero, Julian Milberg, Cassie Bennett, Maria Ines Mitrani, Michael A. Bellio

**Affiliations:** Organicell Regenerative Medicine, Miami, FL, United States

**Keywords:** extracellular vesicles, T cells, immune response, amniotic fluid, inflammation

## Abstract

**Introduction:**

Extracellular vesicles isolated from human amniotic fluid (AF-EVs) have previously been found to modulate inflammation and macrophage infiltration in a mouse model. However, the effects of acellular amniotic fluid (acAF) or AF-EVs on the T-Cell immune response have not been explored.

**Methods:**

In this study, we investigated the effects of acAF and AF-EVs on the T cell immune response in an in vitro cell culture model. Peripheral Blood Mononuclear Cells (PBMCs) were stimulated with Phytohemagglutinin (PHA) to induce the immune response and were subsequently treated with either serum-free media (vehicle), acAF, or concentrated AF-EVs.

**Results:**

Both acAF and AF-EV treatment suppressed PHA-induced T cell proliferation and PHA-induced T cell activation; however, treatment with concentrated AF-EVs had a greater effect. Additionally, both acAF and AF-EVs reduced PBMC pro-inflammatory cytokine release. AF-EVs were found to be taken up by both CD4+ and CD8+ effector T cell subsets.

**Conclusion:**

Overall, this data demonstrates that AF-EVs have a robust immunomodulatory effect on T cells and suggests AF-EVs could be used as an immunotherapeutic tool.

## Introduction

Extracellular vesicles (EVs) are cell derived particles enclosed by a lipid bilayer membrane which play a major role in paracrine signaling by transporting cargo such as RNA and proteins to target cells (1) This has made them a topic of interest for both diagnostic and therapeutic purposes and many preclinical studies have focused specifically on the ability of EVs to combat inflammatory disorders (2–5). EVs can be obtained from *in vitro* cultured cells which release EVs into the cell culture media or from bodily fluids such as blood, urine, and amniotic fluid (AF).

AF is a rich source of EVs that have been characterized to express EV specific tetraspanins CD9, CD63, and CD81, and to carry immune modulatory and anti-inflammatory RNA and protein cargo (6, 7). AF-derived EVs (AF-EVs) can regulate angiogenesis and are capable of altering lung tissue structure (6, 7). Additionally, AF-derived treatments have recently entered the clinic as a novel therapeutic for the treatment of COVID-19 long haulers and COVID-19 induced acute respiratory distress syndrome and inflammation (8, 9). However, the extracellular components of AF have not been extensively studied to understand their biological functions, systemic effects, or therapeutic potential. Instead, multiple studies have focused on studying extracellular secretions and EVs derived from cultured human AF stem cell sources (AFSC-EVs).

AFSC-EVs have been shown to have immunomodulatory and anti-inflammatory properties *in vivo* in various mouse models and offer therapeutic potential for various diseases (10) For example, in a mouse model of skeletal muscle atrophy, mice treated with AFSC-EVs converted macrophages from pro-inflammatory (M1) to anti-inflammatory (M2) phenotypes and attenuated the maturation of memory B cells (11). In a mouse model of allogeneic skin transplantation, AFSC-EVs reduced T cell proliferation and increased populations of regulatory T cells (12). AFSC EVs have also been shown to have anti-inflammatory and immunomodulatory effects *in vitro* by preventing activation of the inflammasome and hindering IL1β production in THP-1 cells (13).

Although AFSCs exhibit immunosuppressive effects and are promising therapeutics for several diseases, their use in clinical applications is limited to their potential to expand in culture so that sufficient amounts are obtained for a clinical dose (14, 15). As in stem cell therapies, a large yield of EVs may be required in order to achieve potency (16). Even if large populations of AFSCs could be generated in culture, another consideration is that many stem cells lose their differentiation potential and original functions after many passages and tend to become senescent (17). AFSC-EV therapies could face similar challenges to cell therapies since different cell culture methods and longer time periods in culture affect the type of EVs released from stem cells, quantities of EV released and EV mRNA content (16, 18). By isolating EVs directly from the amniotic fluid, cell culture is avoided, allowing for a method of manufacturing EVs which does not depend on cell proliferation, cell function, or cell senescence and can be executed in a shorter time frame.

AF-EVs consist of EVs secreted from various cell sources in the surrounding perinatal environment, including AFSCs, but also placental cells (7). Perinatal cells from various tissues and their extracellular vesicles have been shown to suppress immune cell activity in both innate and adaptive immune systems (19). The amnion is the tissue lining of the placenta which is exposed to the amniotic fluid and facing the fetus. Cells from placental amniotic tissue specifically have been shown to prevent B cell differentiation into plasma cells (20). Additionally, amniotic tissue derived cells, or their extracellular secretions, have been shown to suppress T cell proliferation, reduce T cell inflammatory cytokine secretion, and prevent T cells from differentiating into Th1 and Th7 subsets (21–23). Since AF contains EVs from both AFSCs and placental cells, AF-EVs may inherit a combination of immunomodulatory potential from these different perinatal cell types.

Surprisingly, to the author’s knowledge no current publications exist which have studied the effects of acellular AF (acAF) or AF-EVs on immune cell activity. While amniotic fluid-derived biologics are beginning to provide therapeutic potential in and out of the clinic (10, 15), the mechanisms of action for these therapeutics are still not understood. The composition of acAF includes the naturally inherent extracellular components present in full-term amniotic fluid, such as EVs, secreted proteins, and extracellular matrix. In these studies, we investigated the immunomodulatory effects of acAF on lymphocytes *in vitro*. We demonstrate here that acAF and, even more so, AF-EVs have a suppressive effect on T cell proliferation, T cell activation, and pro-inflammatory cytokine release.

## Methods

### AF collection

Human AF was donated from consenting adults during routine, planned cesarean sections under an IRB approved protocol (IRB approval agency: IRCM) in the operating room by aspiration. Donor qualification was performed under FDACFR 1271 guidelines. Donor qualification was certified following the review of the mother’s medical history, social history, physical examination, and raw product recovery information. Relevant communicable disease testing was completed, and the mother was reported to have negative/non-reactive results for CMV IgM Ab, Hepatitis B core total Ab, Hepatitis B surface Ag, Hepatitis C virus Ab, HIV-1/HIV-2 Plus O, HTLV I/II Ab,Syphilis screening—non-treponemal, Ultrio Elite HBV, UltrioElite HCV, Ultrio Eliter HIV-1/2, and WNV. All patients provided preoperative written informed consent. AF collections from 7 donors were used for this study. The average age of donors was 27.71 ± 1.9 years and the average gestational age was 38.3 ± .74 weeks. The average volume of unprocessed AF collected from each donor was 492 ± 140ml.

### acAF and AF-EV preparation

Full-term human amniotic fluid was collected in the operating room by aspiration during planned cesarean sections and shipped to the manufacturing faculty on ice. Upon processing, raw AF was centrifuged at 500g for 10 minutes. The supernatant was collected and centrifuged at 2000g for 20 minutes. The supernatant was then filtered through a.22µm filter to remove all large cell debris and large proteins. The processed product resulted in acAF comprising acellular extracellular components such as EVs and soluble proteins.

A starting volume of 16ml acAF was used for AF-EV preparations. To create the AF-EV preparation, acAF was further centrifuged at 100,000g for 3 hours at 4°C using an Optima XPN-90 Ultracentrifuge (Beckman Coulter) with a type 50.4 Ti fixed-angle titanium rotor (Beckman Coulter). The supernatant was removed, and the pellet was washed by resuspending in sterile DPBS and centrifuging a second time at 100,000g for 2 hours at 4°C. The final EV pellet was resuspended in 500µl DPBS and stored at -80°C until use.

EV-depleted supernatant was prepared by collecting acAF supernatant from the first centrifugation of AF-EV preparations, and centrifuging it at 100,000g for 16 hours. The EV-depleted supernatant was then collected.

### PBMC collection and cell culture

Human peripheral blood mononuclear cells (PBMCs) were isolated from the blood of healthy consenting adult donors using a Ficoll density gradient (Ficoll Paque Plus, Cytiva). 6 donors participated in this study, and 60ml of blood was collected per donor. Blood was centrifuged at 1000g for 15 minutes in 50ml LeucoSep tubes. 1-1.2 million cells were seeded per well in a 24 well plate in RPMI media with 10% FBS and 1% Penicillin/Streptomycin per well in a total volume of 1ml. For PHA-stimulation 5µg/ml of Phytohemagglutinin-M (PHA) (Sigma 11082132001) was added to PBMCs in culture to stimulate T cell proliferation and activation. PBMCs were suspended in 1ml total volume which consisted of 800µl of media and 200µl of a treatment solution or a vehicle control. Treatment solutions added to PHA-stimulated cells included acAF, AF-EV preparations, and EV-depleted supernatant.

### T-cell proliferation

For T cell proliferation experiments PBMCs were stained with CellTrace™ CFSE dye (ThermoFisher Scientific C34554) prior to culturing them. CellTrace™ CFSE dye was diluted 1:1000 in PBS and warmed to 37°C. Diluted CFSE dye was added to PBMCs and incubated at 37°C for 20 minutes. RPMI media with 10% FBS was added to PBMCs and incubated at 37°C for 3-5 minutes to quench the remaining dye.

For the inital T cell proliferation experiments with acAF, to obtain a 10% concentration of acAF, 100µl of serum-free media and 100µl acAF were added to 800µl of media. To obtain a 20% concentration of acAF, 200µl of acAF was added to 800µl of media. 10% and 20% concentrations of acAF were added to experimental treatment groups in addition to the PHA to determine effective dose. An equal volume of serum-free media was added to the PHA only group and the non-stimulated control group. The media was changed at 4 days and was replaced with the same conditions. T cell proliferation was measured 4 days and 8 days after the addition of PHA, treatments, and control.

For the inital experiments with high concentrations of AF-EVs and acAF, the acAF was added at a 20% concentration (200µl in 800µl of media) and contained 4x10^10^ particles per 1x10^6^ cells, while the AF-EV solution was added at a 20% concentration (200µl in 800µl of media) and contained 1x10^11^ particles per 1x10^6^ cells. An equal volume of serum-free media was used for PHA only group. T cell proliferation was measured 5 days after the addition of PHA, treatments, and control. Based on experience with initial experiments, an additional day was added to the early proliferation time point (day 4) to further amplify the percentage of proliferating cells responding to PHA and for the observation of immunosuppressive effects on a larger proliferating cell population at day 5. For T-Cell proliferation experiments, each PBMC donor was performed in triplicate wells and then averaged to create a singular data point.

### T-cell activation

For initial T cell activation experiments with acAF, (200µl in 800µl of media) were added to experimental treatment groups in addition to the PHA. An equal volume of serum-free media was used for the PHA only group and the non-stimulated control group. T cell activation markers CD25 and CD69 were analyzed 1 day and 3 days after the addition of PHA, treatments, and control.

For inital experiments with high concentrations of AF-EVs and acAF, was added at a 20% concentration (200µl in 800µl of media) and contained 4x10^10^ particles per 1x10^6^ cells, while the AF-EV solution was added at a 20% concentration (200µl in 800µl of media) and contained 1x10^11^ particles per 1x10^6^ cells. An equal volume of serum-free media was used for the PHA only group and non-stimulated control group. T cell activation was analyzed 3 days after the addition of PHA, treatments, and control. For T-Cell proliferation experiments, each PBMC donor was performed in triplicate wells and then averaged to create a singular data point.

### acAF and AF-EV dose preparations

For dose response experiments, acAF and AF-EV preparations were diluted in PBS to obtain various nanoparticle doses: 4x10^9^, 2x10^10^, and 4x10^10^. First, the AF-EV preparations were diluted in PBS to obtain an equal concentration of nanoparticles/ml as the undiluted acAF (approximately 2x10^11^ nanoparticles/ml)to create the first stock solutions for the 4x10^10^ dose. To create the other dose stock solutions, the first stock solutions of acAF and AF-EVs were each diluted 1:2 in PBS to obtain the second stock solution for the 2x10^10^ dose and 1:10 in PBS to obtain the third stock solution for the 4x10^9^ dose. Next, the volume of undiluted acAF which contained 4x10^10^ particles (175-200µl) was added to the media. Next, an equal volume of the AF-EV preparation first stock solution was added to the media to match the same nanoparticle dose. Next, an equal volume of second stock solution was added to the media to deliver 2x10^10^ particles, and an equal volume of third stock solution was added to the media to deliver 4x10^9^ particles. Lastly, an equal volume of supernatant was added to the media. All groups were treated with 5µg/ml PHA and an equal volume of PBS was added to the PBS group as a vehicle control. The proliferation studies were completed as described above and proliferation was measured at day 5. T-Cell activation studies were completed as described above and activation markers were measured on day 3.

### Flow cytometry

Cells were collected at different time points, washed with PBS, and centrifuged at 350g for 5 minutes. Cells were resuspended in FACs buffer (Thermo Fisher Scientific) and incubated in antibodies for 25 minutes in the dark at room temperature. Following incubation, cells were washed twice with FACs buffer and analyzed with a Cytoflex (Beckman Coulter). 10000 events were recorded per sample. To analyze T cell proliferation and activation within the PBMC cultures, first CD3+ cells were gated. For T cell activation CD25+ and CD69+ cells were gated within the CD3+ population. For T cell proliferation experiments a CD3 APC (BD Biosciences 555342) antibody was used. For T cell activation experiments CD3 APC (BD Biosciences 555342), CD69 PE (BD Biosciences 654975), and CD25 FITC (BD Biosciences 555431) antibodies were used. For T cell viability experiments CD3 APC (BD Biosciences 555342) and Propidium Iodide PE (Thermo Scientific BMS500PI) antibodies were used. For EV uptake experiments CD3 APC (BD Biosciences 555342), CD4 APC (Biolegend 317415), CD8 APC (Biolegend 344721) antibodies were used. Isotype antibodies used were: Anti IgG1κ PE (BD Biosciences 555749), Anti IgG1 FITC (Thermo Fisher GM4992), Anti IgG1κ APC (BD Biosciences 555751), Anti IgG2aκ APC (BD Biosciences 555576).

### BCA assay

Protein concentrations were calculated using a BCA Assay (abcam). 50µl of each sample was used and samples were run in duplicate. Samples were read on a microplate reader (Molecular Devices) at an absorbance wavelength of 562nm.

### Nanosight

Nanoparticle tracking analysis of both acAF and the EV preparation were performed using a NanoSight NS300 instrument (NTA 3.4 Build 3.4.003). 10 µL of sample was diluted in 10 mL of Cell Culture Grade Water. The capture settings were modified to capture 5 video files with a capture duration of 30 seconds, using a camera level of 15, and a continuous syringe pump flow rate of 50. After completion of the script, the video files were analyzed with a detection threshold of 3. NTA post acquisition settings were kept constant between samples. The size and concentration profiles of each mode were then imported into Prism (Graph Pad, version 9.2.0) and superimposed.

### Fluorescent NTA

Fluorescent NTA (fNTA) was performed using a ZetaView QUATT with ZetaView software version 8.05.12 SP1 (Particle Metrix GmbH,Inning am Ammersee, Germany). AF-EVs were labeled with anti CD63-Alexa 488 (NBP2-42225AF488) and anti CD81-DyLight 550 (NB100-65805R) (Novus Biologicals, Littleton, CO, USA) by adding 1mL of each fluorescent antibody to 20mL of sample containing isolated EVs. The fluorescently labeled EV samples were then incubated for 2h in the dark on ice. The samples were diluted by mixing deionized water filtered through a 0.2-µm syringe filter with corresponding volumes of sample. The fNTA was performed in scatter mode, 488/500 fluorescent mode and 520/550fluorescent mode. For scatter mode analysis, the ZetaView settings were adjusted to have a sensitivity of 75, shutter speed of100, cycles/positions of 2/11, frame rate of 30, maximum size of 1000, minimum size of 20, track length of 15 and minimum brightness of 20. Fluorescent mode analysis had similar parameters with the exception of an increased sensitivity of 8085. The size and concentration profiles of each mode were then imported into Prism (Graph Pad, version 9.2.0) and superimposed. This experiment was repeated using three independent AF-EV preparations.

### Transmission electron microscopy

AF-EVs were analyzed using a JEOL JEM-1230 electron microscope (JEOL USA, Inc, Peabody,MA, USA) in conjunction with formvar carbon-coated transmission electron microscopy grids (FCF400-Cu; Electron Microscopy Sciences,Hatfield, PA, USA). The copper carbon formvar grids were glow-dis-charged prior to loading an undiluted sample of EVs. The grids were then floated on 10mL of EV sample drop for 15 min, washed two times with water by floating on the drop of water for 30 s and then negatively stained with 2% uranyl acetate by floating on the drop of stain for 30 seconds. The grids were blot-dried with Whatman paper (Cytiva, Marlborough, MA, USA) and then imaged.

### Capillary western blot

Unprocessed (raw) AF lysate was prepared by centrifuging unprocessed amniotic fluid at 1000g for 10 min to pellet cell debris and proteins. The pellet was lysed in 0.2mL RIPA buffer and centrifuged at 14,000g for 10 minutes. Protein was measured with a BCA assay for both raw AF lysate and AF-EV samples and the same amount of protein (80 ug/mL) was loaded for each sample. Capillary western was performed using 12-230 kDa prefilled plates (Bio-techne) with immunoassay and total protein detection. For total protein analysis a total protein detection module for chemiluminescence based assays (Bio-techne) was used. Separation time was 25 minutes, separation voltage was 375 V, primary antibody incubation time was 40 minutes, and secondary antibody incubation time was 30 minutes. Antibody signal was detected with HDR Chemiluminescence. Antibodies used were CD9 (Cell signaling technologies), TSG101 (Novus Biologicals), Annexin V (R&D Systems), and GM130 (Novus Biologicals).

### Cytokine analysis

200µl of media from PBMCs in culture was collected from each well and diluted 1:2 in serum-free RPMI media. Eve Technologies performed a multiplex immunoassay (Luminex, HDF-15) to analyze pro-inflammatory cytokine levels.

### CFSE staining of EVs

EVs were stained according to protocol described in Morales-Kastresana et al., 2017 with a few modifications (24). CellTrace™ CFSE dye (ThermoFisher Scientific C34554) was diluted 1:100 in PBS and warmed to 37°C. Diluted CFSE dye was added to AF-EV preparation at a 1:1 volume ratio and incubated at 37°C for 2 hours. Diluted CFSE dye was added to PBS at a 1:1 volume ratio as a control and prepared identical to the AF-EV preparation. 0.1% FBS in PBS was added to EV preparation and incubated at 37°C for 3-5 minutes to quench the remaining dye. The EV preparation was filtered through a Amicon Ultra-4 Centrifugal Filter Unit with Ultracel-100 membrane-100 kDa MWCO by centrifuging at 4000g for 15 minutes. The remaining EV solute was washed once with PBS and centrifuged at 4000g for 15 minutes. For cell culture experiments 1x10^11^ particles from the EV solute were added to each well containing 1x10^6^ PBMCs stimulated with PHA and incubated for 3 days.

### Statistical analysis

Data were expressed as mean± SD. Data points within each figure represent experiments with unique PBMC donors (n=3). The number of acAF, AF-EV, and AF-supernatant donors used within each experiment is indicated in the figure legends. Statistical analysis was performed by Prism software (GraphPad version 9.3.1) using one-way ANOVA with Tukey’s Test for multiple comparisons. Student’s t-test was used to analyze T cell viability. P<0.05 was considered statistically significant. The cell response to PHA stimulation may vary depending on the PBMC donor, therefore the T cell response was graphed as a percentage of the PHA vehicle control group average within the same experiment. Each experiment in this study was repeated with PBMCs from 3 separate donors.

## Results

### acAF attenuates PHA-induced T cell proliferation and activation

**Figure 1:**
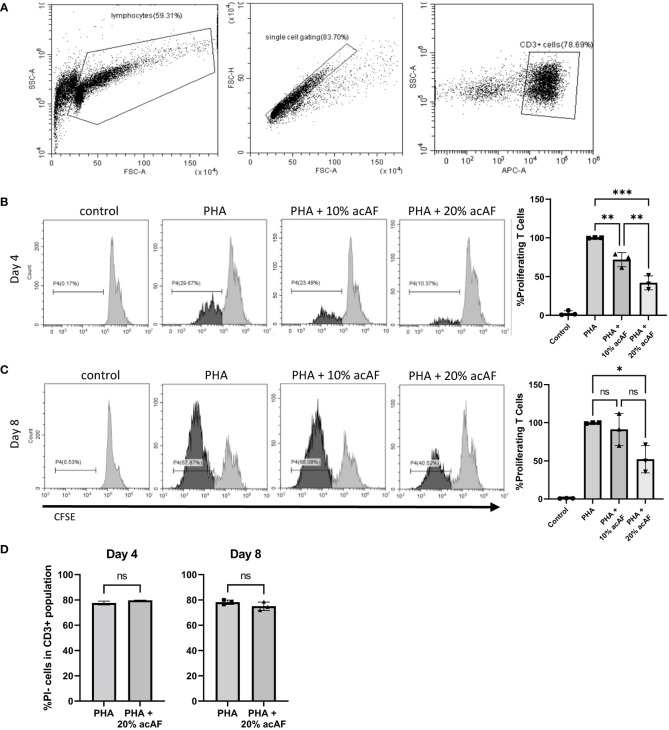
AcAF Attenuates T Cell Proliferation 4 and 8 days after PHA Stimulation **(A)** Representative flow cytometry CD3+ gating. Representative histograms of proliferating CD3+ T cells in each group and graphed as percentages of the PHA vehicle control group following **(B)** 4-day PHA stimulation or **(C)** 8-day PHA stimulation. **(D)** Graphed percentages of PI- cells within CD3+ population. (Data graphed as mean ± SD; One way ANOVA with Tukey’s test; n=3 for each group; *=p<.05; **=p<.01; ***=p<.001; ns=not significant; Data is derived from 3 separate experiments. Each experiment used 1 independent PBMC donor treated with 1 of 3 acAF donors).

To study the immunomodulatory effects of acAF *in vitro*, PBMCs were stimulated with PHA to induce cell proliferation for up to 8 days. 4 days following PHA stimulation, acAF suppressed T cell proliferation with both 10% and 20% concentrations (**Figure 1B**). Treatment with 10% acAF reduced the proliferating T cell population to 72 ± 9% of the PHA vehicle control group (p<0.01), while treatment with 20% acAF reduced the proliferating T cell population to 42 ± 9% of the PHA vehicle control group (p<0.001). There was no significant change in T cell proliferation in the group treated with 10% acAF treatment at the 8 day time point (**Figure 1C**). However, treatment with 20% acAF reduced the T cell proliferating population to 52 ± 17% of the PHA vehicle control group (P<0.05), suggesting a larger dose of acAF is required to reduce T cell proliferation over a longer time course. T cell viability was not affected by 20% acAF treatment at either time point (**Figure 1D**). A 20% concentration of acAF treatment was used for all further experiments since this concentration was found to be most effective.

**Figure 2:**
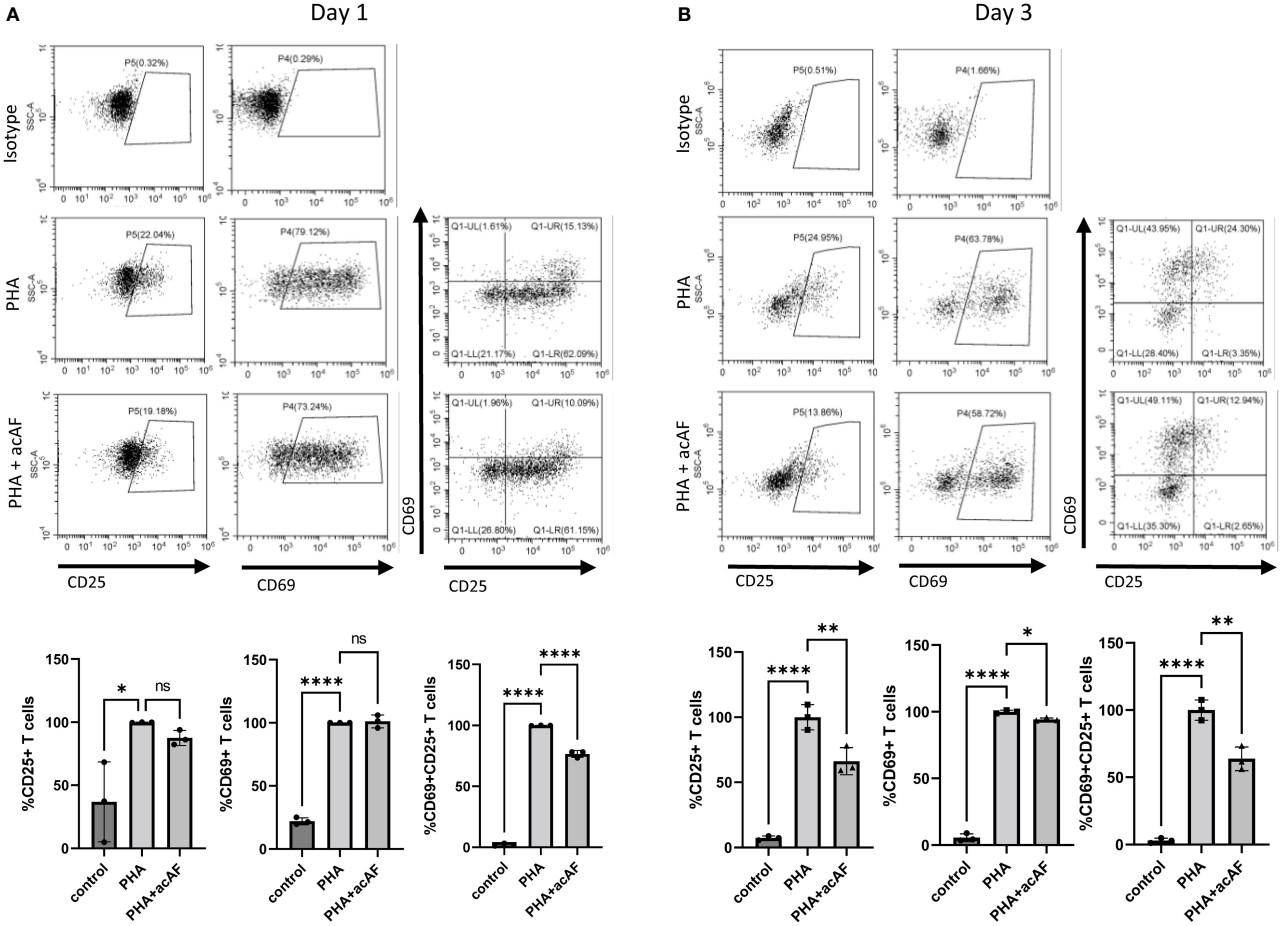
AcAF Attenuates T Cell Activation 1 and 3 days after PHA Stimulation CD69+ and CD25+ cells were gated from CD3+ T cell population. Cells expressing CD69, CD25, and both CD69 and CD25 were analyzed within T cell population following **(A)** 1-day PHA stimulation and **(B)** 3-day PHA stimulation in each group and graphed as percentages of the PHA vehicle control group. (Data graphed as mean ± SD; One way ANOVA with Tukey’s test; n=3-4 replicates for each group; ns=not significant; *=p<.05; **=p<.01; ****=p<.0001; Data is derived from 3 separate experiments. Each experiment used 1 independent PBMC donor treated with 1 of 3 acAF donors).

To further explore immunomodulatory effects of acAF on T cells, the same cell culture model was used to study T cell activation. Upon activation, T cells overexpress membrane bound receptors CD69 and CD25. CD69 is an early-stage activation marker while CD25 is a later stage activation marker and has high expression levels about 2 days following activation until about 4-5 days (25). After 1 day of stimulation with PHA, there was a significant increase in both CD25+ and CD69+ T cells compared to non-stimulated control cells (p<0.0001) (**Figure 2A**). acAF reduced the population of activated T cells expressing both CD25 and CD69 (p<0.0001) but did not significantly change the populations of T cells expressing either CD25 or CD69 at this time point. After 3 days of stimulation with PHA, acAF treatment significantly reduced the CD25+ T cell population (p<0.01), the CD69+ T cell population (p<0.05), and the CD25+CD69+ T cell population (p<0.01) in PHA stimulated cells at the 3-day time point (**Figure 2B**). Overall, acAF treatment significantly reduced T cell activation and had a greater effect at later stages of T cell activation.

### AF-EV characterization

**Figure 3:**
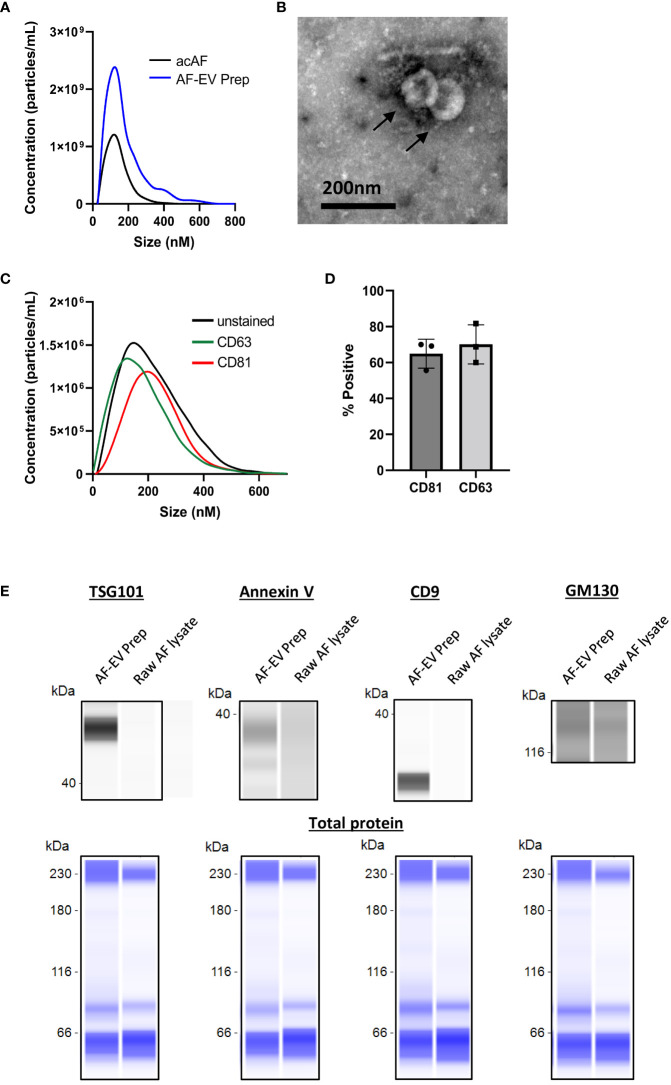
Characterization of AF-EVs **(A)** Comparison of nanoparticle populations between AcAF and EV Preparation samples **(B)** Transmission electron microscopy image of vesicles within EV preparation **(C)** Representative CD81+ and CD63+ particle populations in relation to total particle population of EV preparation **(D)** Percentage of particles in EV preparation expressing exosomal markers CD81 and CD63 (Data graphed as mean ± SD; n=3 samples). **(E)** Representative capillary western blots of EV markers (TSG101, Annexin V, and CD9) and of cellular Golgi body marker (GM130) in AF-EV Preparation samples and in raw, or unprocessed, AF lysate samples (top). The same amount of protein was loaded for each sample and total protein blots are shown in blue below each corresponding sample to visualize total loaded protein (bottom).

To investigate whether EV mechanisms are involved in acAF’s immunomodulatory effects on T cells, small EVs were further isolated from acAF using ultracentrifugation to create a concentrated EV preparation. Amniotic fluid contains high quantities of EVs and our group has previously published extensive characterization work for AF-EVs prepared with this isolation method (6) Protein concentrations were measured and particle analysis was performed for both acAF and AF-EV preparation samples. The small EV yield within the EV preparation was 1.61 x10^10^ particles/ml of unprocessed AF. The EV preparation had relatively low protein contamination (0.14 ± 0.03mg/ml), while the acAF sample had greater concentrations of protein (2.89 ± 0.26mg/ml). The EV preparation had over twice the concentration of particles as the initial acAF product (4.3 x10^11^ particles/ml compared to 1.8x10^11^ particles/ml in acAF); however, there was little variation in particle size between samples, with the mode particle sizes ranging from 135 to 172nM (**Figure 3A**).

AF-EVs were further characterized with TEM and fNTA. Transmission electron microscopy identified vesicles with a round morphology with an approximate size of 100nm (**Figure 3B**). fNTA analysis revealed the presence of exosome-associated transmembrane proteins CD81 and CD63 in the EV preparation. AF-EV nanoparticles were 64.9 ± 8.1% positive for CD81 and 70.1 ± 10.9% positive for CD63 (**Figures 3C, D**). Capillary western blot analysis confirmed the expression of EV marker proteins TSG101, Annexin V, and CD9 in the AF-EV preparation samples only, and relatively weak expression of Golgi body marker GM130 in both the AF-EV sample and raw AF lysate sample (**Figure 3E**). The raw AF lysate is not a pure cell product (it contains a mixture of vernix caseosa, extracellular matrix, proteins, and debris); therefore, cell organelle marker expression may be lower in this sample compared to pure cell lysate samples. Nonetheless, these results confirm our AF-EV preparation is enriched with EV positive markers compared to AF starting material.

### AF-EVs exert immunomodulatory effects on T cells

To test the effects of AF-EVs on T cell proliferation and activation, a high concentration of AF-EVs (1x10^11^ particles) from the EV preparation were added to PHA-stimulated PBMCs as an AF-EV treatment. In comparison, acAF treated wells had approximately 4x10^10^ particles added to each well. AcAF treated wells had approximately 0.3mg of protein added to each well while AF-EV treated wells had approximately 0.03mg of protein.

**Figure 4:**
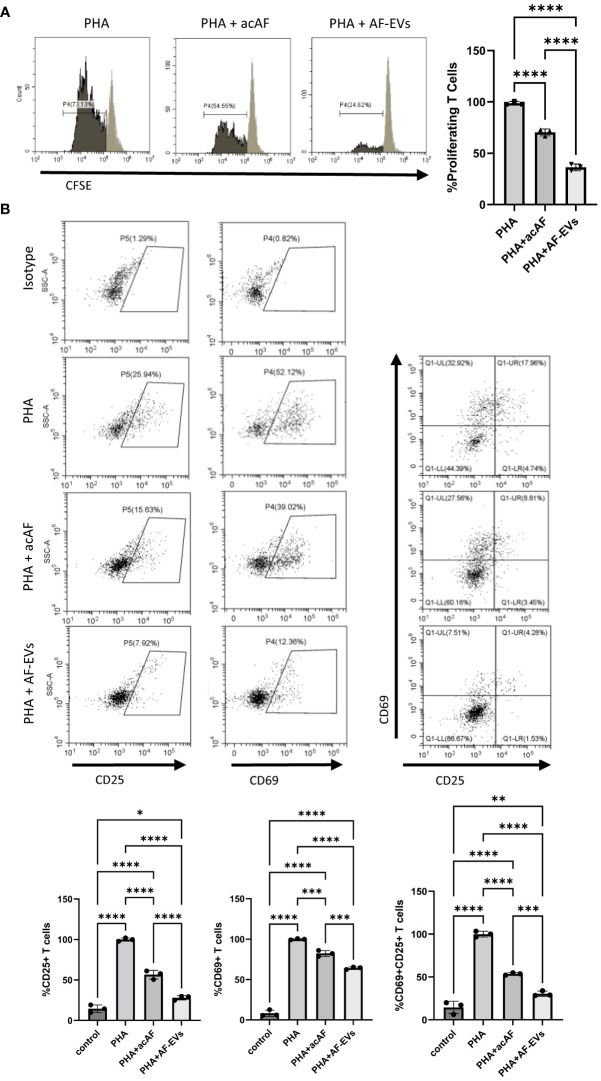
Concentrated AF-EVs Have a Greater Effect on T Cell Proliferation and T Cell Activation than acAF **(A)** Representative histograms and proliferating CD3+ T cells in each group graphed as percentages of the PHA vehicle control group after 5 days of culture **(B)** CD69+ and CD25+ cells were gated from CD3+ T cell population. Percentages of cells expressing CD69, CD25, and both CD69 and CD25 were analyzed within T cell population in each group and graphed as percentages of the PHA vehicle control group after 3 days of culture. (Data graphed as mean ± SD; One way ANOVA with Tukey’s test; n=3-4 for each group; *=p<.05 **=p<.01; ***=p<.001; ****=p<.0001; Data is derived from 3 separate experiments. Each experiment used 1 independent PBMC donor treated with 1 of 2 acAF and 1 of 2 AF-EV donors).

Concentrated AF-EVs had a more robust effect on both T cell proliferation and T cell activation than acAF. Both acAF and AF-EV treatment significantly reduced proliferation within the T cell population after 5 days of treatment (p<0.0001) (**Figure 4B**). However, AF-EV treatment suppressed T cell proliferation significantly more than acAF treatment (p<0.0001). acAF treatment reduced the proliferating T cells to 70 ± 3% of the PHA vehicle treated group, while the AF-EV treatment reduced the proliferating T cells to 36 ± 2% of the PHA vehicle treated group.

Additionally, concentrated AF-EVs reduced T cell activation significantly more than acAF treatment in CD25+ (p<0.0001), CD69+ (p<0.001), and CD25+CD69+ (p<0.001) cells (**Figure 4B**). The acAF treatment reduced the population of CD25+ T cells to 56 ± 5% of the PHA vehicle control group, and the AF-EV treatment reduced the population of CD25+ T cells to 28 ± 3% of the vehicle control group. The acAF treatment reduced the population of CD69+ T cells to 82 ± 4% of the PHA vehicle control group, and the AF-EV treatment reduced the population of CD69+ T cells to 64 ± 2% of the vehicle control group. The acAF treatment reduced the population of CD25+CD69+ T cells to 54 ± 1% of the PHA vehicle control group, and the AF-EV treatment reduced the population of CD25+CD69+ T cells to 30 ± 3% of the vehicle control group. There was a small difference between the CD25+ T cell populations in the AF-EV treated group and the non-stimulated control group (p<.05) compared to the difference between vehicle treated group and the non-stimulated control group (p<0.0001), suggesting concentrated AF-EV treatment maintained or restored CD25 expression to levels similar to those of naive T cells.

**Figure 5:**
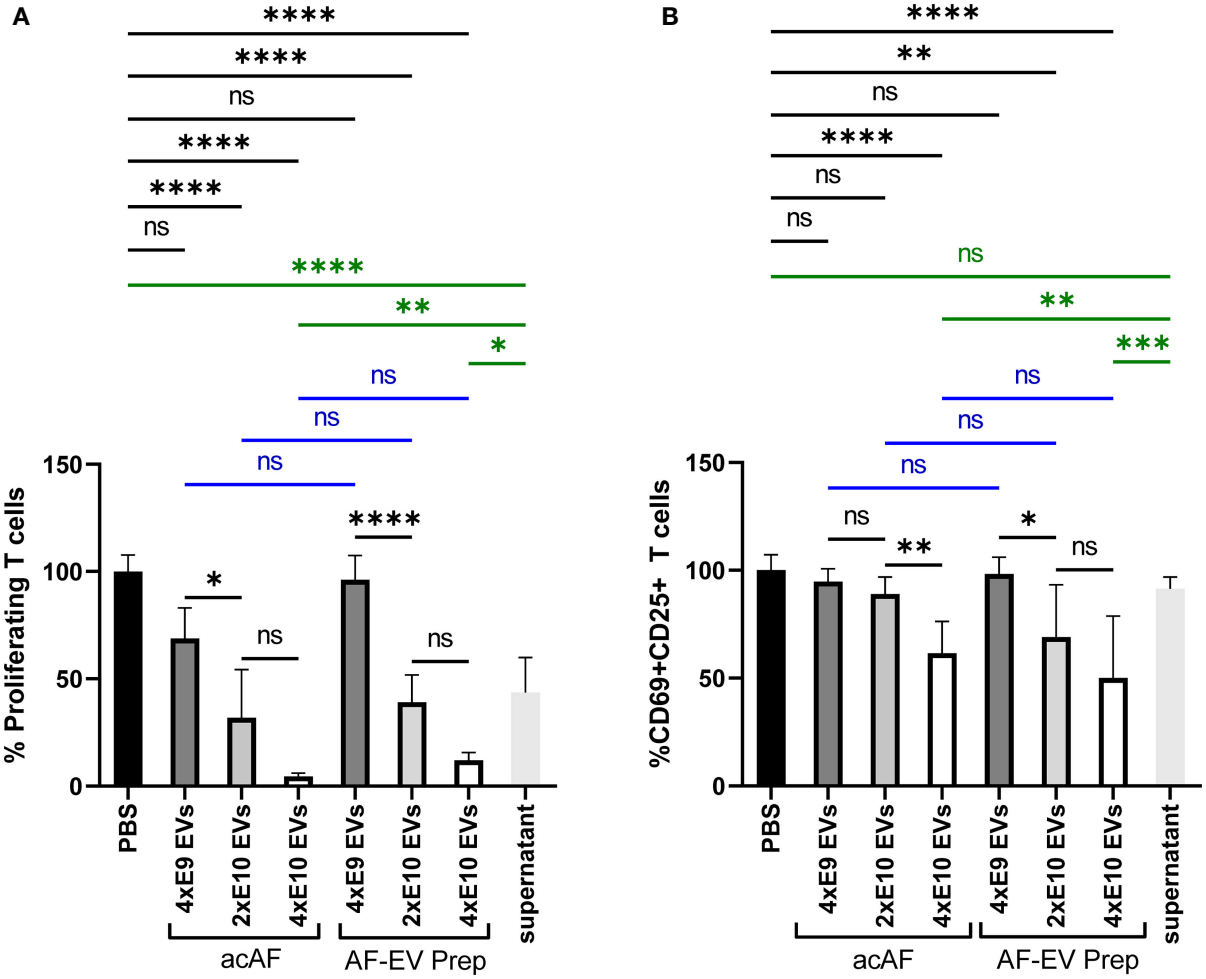
AF-EVs Have a Dose-Dependent Suppressive Effect on T Cell Proliferation and T Cell Activation PBMCs were stimulated with PHA and treated with either PBS, doses of acAF, doses of AF-EV Preparation, or EV-depleted acAF supernatant **(A)** Proliferating T cells treated with PBS or different doses of acAF or AF-EV Preparation following PHA stimulation were graphed as a percentage of proliferating T cells in PBS treated control group. **(B)** CD69+CD25+ T cells treated with PBS or different doses of acAF or AF-EV Preparation following PHA stimulation were graphed as a percentage of CD69+CD25+ T cells in PBS treated control group. (Data graphed as mean ± SD; One way ANOVA with Tukey’s test; Blue= equivalent dose comparisons between acAF and AF-EV Prep; Green= comparisons with EV-depleted supernatant; n=3 for each group; ns=not significant; *=p<.05; **=p<.01; ***=p<.001; ****=p<.0001; Data is derived from 3 separate experiments. Each experiment used 1 independent PBMC donor treated with 1 of 3 acAF donors, 1 of 3 AF-EV donors, and 1 of 2 EV-depleted supernatant donors.).

To further study the effects of AF-EVs on T cell function, a dose response was measured for both acAF and AF-EV treatment on PHA-stimulated PBMCs. Equivalent doses of nanoparticles from acAF and AF-EV preparations were added to compare the effects from each and to determine if other components of acAF other than EVs may be having an effect. EVs had a dose-dependent effect in both T cell proliferation and T cell activation (**Figure 5**). There was no significant difference between the effects of acAF and AF-EV preparations with equivalent doses on T cell proliferation (**Figure 5A**). When the EV-depleted supernatant from acAF was added to PHA-stimulated PBMCs, this treatment also suppressed T cell proliferation, but this effect was less suppressive than that of the acAF (**Figure 5B**). There was no significant difference between the effects of acAF and AF-EV preparations with equivalent EV doses on T cell activation, and the acAF EV-depleted supernatant had no effect on T cell activation (**Figure 5**). This suggests there may be a component in the acAF other than EVs which affects T cell proliferation, but not T cell activation.

### AcAF and AF-EVs reduce extracellular pro-inflammatory cytokine levels

**Figure 6:**
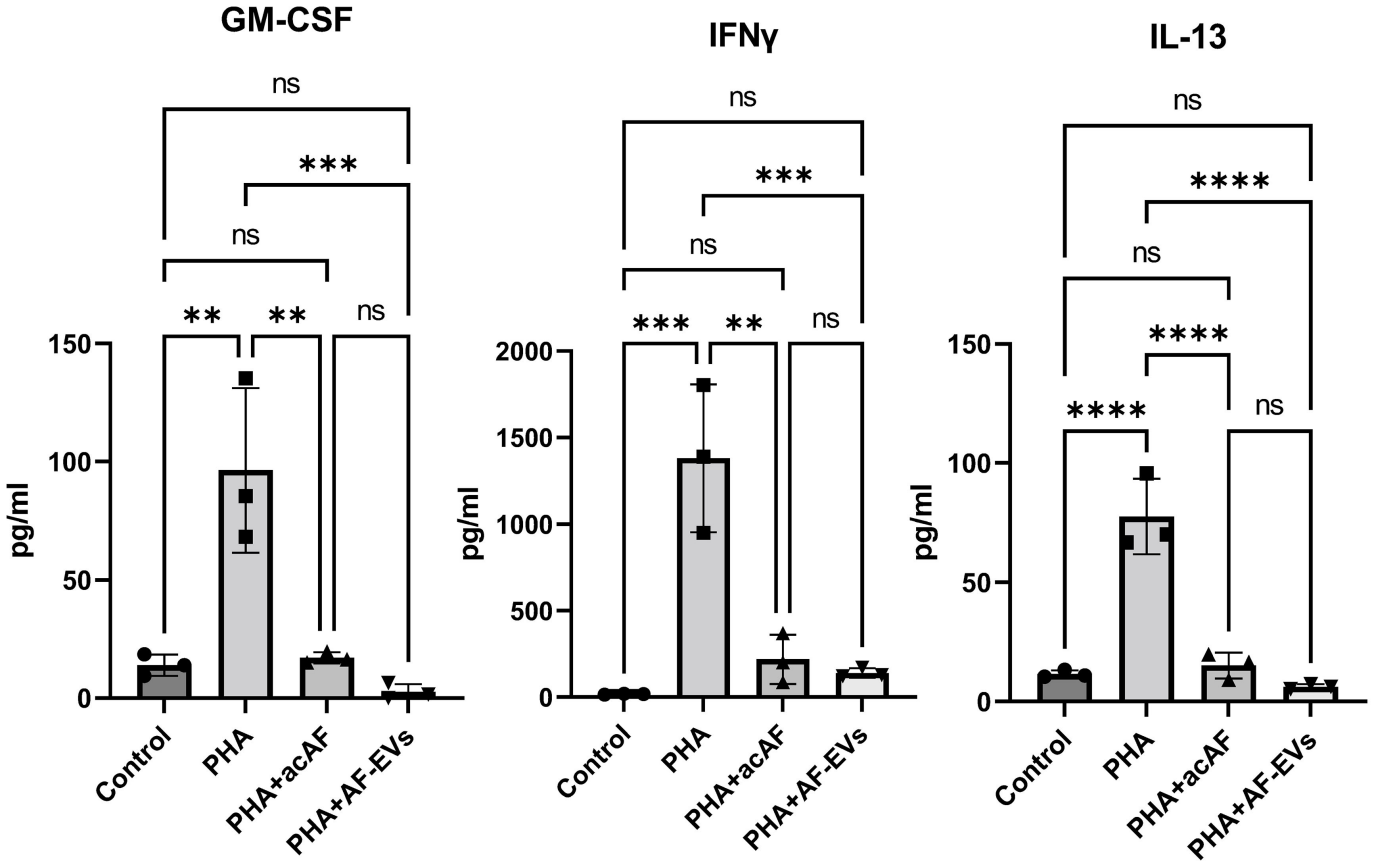
AcAF and AF-EV Treatment Reduce PBMC Pro-Inflammatory Cytokine Release Extracellular levels of pro-inflammatory cytokines released into media were measured after 3 days of culture (Data graphed as mean ± SD; One way ANOVA with Tukey’s test; n=3 for each group; **=p<.01; ***=p<.001; ****=p<.0001; ns=not significant. Data is derived from 3 separate experiments. Each experiment used 1 independent PBMC donor treated with 1 of the 2 acAF and 1 of the 2 AF-EV donors).

To further study the effects of acAF and AF-EVs on immune cell activity, we analyzed pro-inflammatory cytokine release from PBMCs stimulated with PHA. PHA stimulation increased pro-inflammatory cytokine secretion from PBMCs, and both acAF and AF-EV treatment strikingly decreased PHA-induced release of pro-inflammatory cytokines GM-CSF, IFNγ, and IL-13 (**Figure 6**). acAF and AF-EV treatment decreased extracellular GM-CSF levels by 80 ± 5% (p<0.01) and 98 ± 2% (p<0.001) respectively, IFNγ levels by 83 ± 11% (p<0.01) and 88 ± 5% (p<0.001), and IL-13 levels by 80 ± 7% (p<0.0001) and 92 ± 3% (p<0.0001). There was no significant difference between acAF and AF-EV treatment; however, average levels of all three pro-inflammatory cytokines were slightly lower with AF-EV treatment than with acAF. There was also no significant difference between control and PHA-stimulated cells treated with acAF or AF-EVs, indicating treatment restored extracellular pro-inflammatory cytokines close to non-stimulated baseline levels.

### AF-EVs are taken up by T effector cells

**Figure 7:**
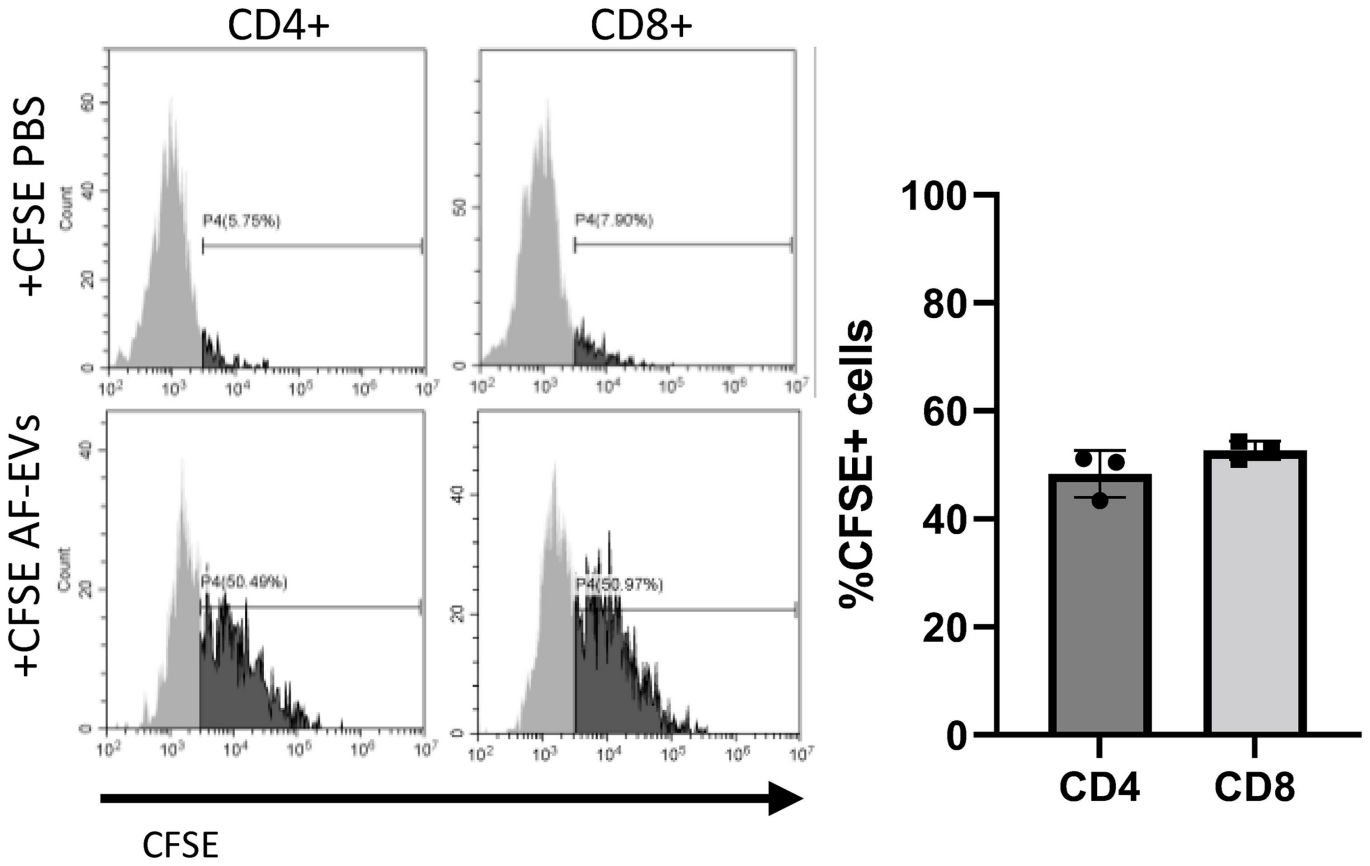
AF-EV uptake in T effector cells. Representative histograms and graphed percentages of CFSE labeled cells within each cell population. CFSE labeled cells were analyzed after 3 days of culture with CFSE EVs (Data graphed as mean ± SD; n=3 for each group; Each experiment used 1 independent PBMC donor treated with 1 of 3 AF-EV donors).

To investigate whether AF- EVs were being taken up by T cells, AF-EVs were labeled with CFSE and added to PHA-stimulated PBMCs. EV uptake was measured in T helper cells (CD4+) and cytotoxic T cells (CD8+) since these are the main subsets of T cells involved in activating the immune response. Both subsets of T effector cells, CD4+ and CD8+, exhibited AF-EV internalization (**Figure 7**). AF-EVs were taken up in 48 ± 4% of CD4+ cells and 58 ± 2% of CD8+ cells after 3 days in culture. This suggests AF-EVs have a direct effect on T effector cells following internalization, rather than acting on T effector cells only indirectly *via* other cell types.

## Discussion

Extracellular vesicle-based therapies are emerging as the next generation of cell-derived therapies for a variety of inflammatory-related diseases including respiratory, cardiovascular, neurological and autoimmune disorders (26–30). EV therapies have demonstrated systemic anti-inflammatory effects in various disease models and can alter phenotypes of different immune cells including macrophages, microglia, neutrophils, B cells, and T cells (2). Therefore, understanding the effect of EVs on T-cell function in an isolated *in vitro* model is of interest to unveil the mechanism of action behind EV therapeutics that have systemic effects on the immune system and inflammation.

Here we demonstrate for the first time that acAF treatment affects immune cell function by altering T cell proliferation and activation following stimulation *in vitro*. AF contains various proteins, including growth factors and anti-inflammatory cytokines IL1ra, IL-10 and IL-12 (31–33); which could presumably activate cell signaling pathways and modulate immune cell activity (34). The data presented here suggests that AF’s immunomodulatory effects on T cells are more dependent on EV mechanisms rather than on free-floating proteins found outside of vesicles within the AF since higher concentrations of AF-EVs had a greater effect on T cells than higher concentrations of AF proteins in acAF (**Figures 3**, **4**). Additionally, acAF had a greater effect on T cell proliferation than the EV-depleted supernatant from acAF, and the EV-depleted supernatant had no effect on T cell activation (**Figure 5**). Additionally, there was greater suppressive effect on T cell proliferation and T cell activation with higher doses of AF-EVs, even in the absence of the acAF supernatant (**Figure 5**). Together this data implies AF-EVs are a main source of immunomodulatory potential in the acAF, yet does not negate a potential supplemental effect from soluble factors.

We have demonstrated that the AF-EV preparation had a higher concentration of particles and most of the proteins from the acAF were removed. Additionally, we demonstrated the majority of the particles were positive for EV-specific markers (**Figure 3**). However, one limitation to this study is that the EV preparation may still contain co-isolated proteins and nanoparticles which were not removed by filtration or ultracentrifugation which could possibly affect AF-EV or T cell function. Nonetheless, this particular AF-EV enriched preparation, which may include co-isolated proteins, has a robust effect on T cells *in vitro* which could translate to a therapeutic effect in the clinic.

To determine if there are specific miRNAs or proteins within the AF-EV cargo that are exerting these immunomodulatory effects on T cells, future work may focus on studying different components found within the AF-EV cargo separately. Since AF-EVs are taken up by T effector cells (**Figure 7**), they are presumptively releasing cargo within the cells that affects cell function. Preliminary characterization studies on the AF-EVs have identified various proteins and miRNA that may be targets of interest. Proteomic analysis of AF-EV content has revealed a high proportion of AF-EV cargo proteins are related to inflammatory diseases and disorder functions (6). Additionally, immune cell trafficking and humoral immune response were within the top physiological functions related to AF-EV cargo proteins. Highly expressed miRNAs within the EV cargo have also been tied to immune related functions (6). The most highly expressed miRNA within AF-EV cargo was let7b, which hampers certain bacterial induced immune responses and inflammation by targeting TLR4 pathways (35). This suggests specific proteins or miRNA within the EV cargo could be altering T cell immune responses.

Many studies have evaluated the immunosuppressive effects of stem cell-derived EVs isolated from cell culture medium (5); however, the novel findings in this study elucidate how EVs isolated from a biological fluid such as AF are similarly capable of immunosuppressive effects. EVs derived from mesenchymal stem cells (MSCs) have been shown to modulate various types of immune cell activity. MSC EVs can suppress proliferation and cytotoxicity of NK cells and inhibit activation of dendritic cells (36, 37). EVs derived from bone marrow MSCs can induce T cell apoptosis and increase T regulatory: T effector cell ratio ( (38). EVs derived from AF have previously been shown to modulate lung tissue structure and vascularization (6, 7); however, this is the first time AF-EVs have been shown to affect immune cell activity. Together, this data further demonstrates that AF is a promising and novel source for therapeutic EVs and depicts a potential mechanism of action through the ability to alter the T-cell response.

During pregnancy the maternal immune system is altered to suppress the immune response to develop tolerance to proliferating cells from the fetus (39, 40), and some of these immunological changes can be observed within T cell populations. For example, there is a decrease in the overall CD3+ T cell population, CD4+ T cells, and CD8+ T cells during late pregnancy compared to before pregnancy (41, 42). There is also a reduction of PHA induced T cell proliferation in pregnant women compared to non-pregnant controls (43). Many of these immunological changes during pregnancy are mediated through EV signaling within the placenta (41). Therefore, it is not surprising that EVs derived from perinatal tissue or AF would promote immunosuppression under certain environmental conditions. A previous study by another group has demonstrated placenta-derived exosomes suppress T cell proliferation and reduce expression of genes associated with T cell proliferation and activation in monocytes (44). Similarly, our data here demonstrates AF-EVs are internalized by T effector cells and can modulate immunological responses by inhibiting T cell activation and proliferation and reducing anti-inflammatory cytokine release. Furthermore, following PHA stimulation T cell activation marker expression and pro-inflammatory cytokine release in AF-EV treated cells were similar to those of non-stimulated cells (**Figures 4**, **5**), suggesting AF-EVs either prevent T cell activation or restore T cells to their naïve non-stimulated state.

Outside of cell culture studies, both AF-EV and acAF treatment have been shown to have anti-inflammatory effects *in vivo* in animal models and in the clinic. In a neonatal rat model of bronchopulmonary dysplasia, hyperoxia injury led to pulmonary remodeling of the alveolar and vascular structure. Administration of AF-EVs in the early stage of injury, improved lung tissue structure, reduced macrophage infiltration, and reduced expression of pro-inflammatory cytokines: IL1a, IL1b, MCP-1, and MIP-1a (6). Zofin, an acAF biologic containing AF-EVs, is hypothesized to reduce systemic inflammation and is currently being tested in the clinic to treat SARS-COV2 infection and post SARS-COV2 long hauler syndrome [NCT05228899]. Zofin has already been tested on a subset of COVID-19 patients and has shown promising clinical results such as decreases in pro-inflammatory cytokine levels (8, 9, 45). Here we show the potential of acAF to decrease pro-inflammatory cytokine release in stimulated PBMCs, specifically GM-CSF, IL-13, and IFNγ. IL13 would be a promising target for pulmonary inflammation and COVID therapeutics since it is associated with severity of COVID and ventilation requirement in COVID patients ( (46). High levels of IFNγ along with other IFN family members, IFNβ and IFNλ2/3, are strongly associated with long COVID syndrome making them another appealing target for COVID therapies (47).

In conclusion, here we identify the capability of AF-EVs to alter immune cell function by attenuating T cell activity and pro-inflammatory cytokine release. Although acAF has similar effects on T cell proliferation and T cell activation, the benefit of using AF-EVs over acAF is that they can be concentrated through ultracentrifugation or other methods to deliver a stronger effect with smaller volumes. This study highlights the potential to harness EVs from amniotic fluid for use in clinical applications for various diseases or disorders with dysfunctional or hyperactive T cells.

## Data availability statement

The raw data supporting the conclusions of this article will be made available by the authors, without undue reservation.

## Ethics statement

The studies involving human participants were reviewed and approved by an Institutional Review Board (IRB). Written informed consent to participate in this study was provided by the participants.

## Author contributions

TR and MB contributed to conceptualization and design of study. TR and JM executed experiments and analyzed data. TR drafted the manuscript. TR, MB, MM, and CB edited and revised the manuscript. All authors contributed to manuscript revision and read and approved the submitted version.

## Conflict of interest

All authors are employees of Organicell Regenerative Medicine, Inc. MM is the chief science officer, serves on the Organicell Regenerative Medicine Board of Directors and holds equity in the company.

## Publisher’s note

All claims expressed in this article are solely those of the authors and do not necessarily represent those of their affiliated organizations, or those of the publisher, the editors and the reviewers. Any product that may be evaluated in this article, or claim that may be made by its manufacturer, is not guaranteed or endorsed by the publisher.

## References

[1] Tetta C Ghigo E Silengo L Deregibus MC Camussi G . Extracellular vesicles as an emerging mechanism of cell-to-cell communication. Endoc (2013) 44:11–9. doi: 10.1007/s12020-012-9839-0 PMC3726927 23203002

[2] Tang TT Wang B Lv LL Liu BC . Extracellular vesicle-based nanotherapeutics: Emerging frontiers in anti-inflammatory therapy. Theranostics (2020) 10:8111–29. doi: 10.7150/thno.47865 PMC7381724 32724461

[3] Suh JH Joo HS Hong EB Lee HJ Lee JM . Therapeutic application of exosomes in inflammatory diseases. vol. 22. Int J Mol Sci (2021) p:1–22. doi: 10.3390/ijms22031144 PMC7865921 33498928

[4] Harrell CR Jovicic N Djonov V Arsenijevic N Volarevic V . Mesenchymal stem cell-derived exosomes and other extracellular vesicles as new remedies in the therapy of inflammatory diseases. Cells (2019) 8(12):1605. doi: 10.3390/cells8121605 31835680 PMC6952783

[5] Xie M Xiong W She Z Wen Z Abdirahman AS Wan W . Immunoregulatory effects of stem cell-derived extracellular vesicles on immune cells. Front Immunol Front Media S.A (2020) 11. doi: 10.3389/fimmu.2020.00013 PMC7026133 32117221

[6] Bellio MA Young KC Milberg J Santos I Abdullah Z Stewart D . Amniotic fluid-derived extracellular vesicles: characterization and therapeutic efficacy in an experimental model of bronchopulmonary dysplasia. Cytotherapy (2021) 23(12):1097–107.doi: 10.1016/j.jcyt.2021.07.011 34538718

[7] Gebara N Scheel J Skovronova R Grange C Marozio L Gupta S . Single extracellular vesicle analysis in human amniotic fluid shows evidence of phenotype alterations in preeclampsia. J Extracell Vesicles (2022) 11(5):e12217. 10.1002/jev2.12217 35582873 PMC9115584

[8] Mitrani MI Bellio MA Sagel A Saylor M Kapp W VanOsdol K . Case report: Administration of amniotic fluid-derived nanoparticles in three severely ill COVID-19 patients. Front Med (2021) 8doi: 10.3389/fmed.2021.583842. PMC8010176 33816515

[9] Bellio MA Bennett C Arango A Khan A Xu X Barrera C . Proof-of-concept trial of an amniotic fluid-derived extracellular vesicle biologic for treating high risk patients with mild-to-moderate acute COVID-19 infection. Biomater Biosyst (2021) 4:100031doi: 10.1016/j.bbiosy.2021.100031. 34841370 PMC8611818

[10] Costa A Quarto R Bollini S . Small extracellular vesicles from human amniotic fluid samples as promising theranostics. Int J Mol Sci (2022) 23(2):590. doi: 10.3390/ijms23020590. 35054775 PMC8775841

[11] Balbi C Piccoli M Barile L Papait A Armirotti A Principi E . First characterization of human amniotic fluid stem cell extracellular vesicles as a powerful paracrine tool endowed with regenerative potential. Stem Cells Trans Med (2017) 6(5):1340–55doi: 10.1002/sctm.16-0297. PMC5442724 28271621

[12] Romani R Pirisinu I Calvitti M Pallotta MT Gargaro M Bistoni G . Stem cells from human amniotic fluid exert immunoregulatory function *via* secreted indoleamine 2,3-dioxygenase1. J Cell Mol Med (2015) 19(7):1593–605. doi: 10.1111/jcmm.12534 PMC4511357 25783564

[13] Mezzasoma L Bellezza I Orvietani P Manni G Gargaro M Sagini K . Amniotic fluid stem cell-derived extracellular vesicles are independent metabolic units capable of modulating inflammasome activation in THP-1 cells. FASEB J (2022) 36(4):e22218. doi: 10.1096/fj.202101657R. 35218567

[14] Sessarego N Parodi A Podestà M Benvenuto F Mogni M Raviolo V . Multipotent mesenchymal stromal cells from amniotic fluid: Solid perspectives for clinical application. Haematologica (2008) 93(3):339–46doi: 10.3324/haematol.11869. 18268281

[15] Loukogeorgakis SP de Coppi P . Concise review: Amniotic fluid stem cells: The known, the unknown, and potential regenerative medicine applications. Stem Cells (2017) 35:1663–73doi: 10.1002/stem.2553. 28009066

[16] de Almeida Fuzeta M Bernardes N Oliveira FD Costa AC Fernandes-Platzgummer A Farinha JP . Scalable production of human mesenchymal stromal cell-derived extracellular vesicles under serum-/Xeno-Free conditions in a microcarrier-based bioreactor culture system. Front Cell Dev Biol (2020) 8doi: 10.3389/fcell.2020.553444. PMC7669752 33224943

[17] Liu J Ding Y Liu Z Liang X . Senescence in mesenchymal stem cells: Functional alterations, molecular mechanisms, and rejuvenation strategies. Front Cell Dev Biol Front Media S.A (2020) 8doi: 10.3389/fcell.2020.00258. PMC7232554 32478063

[18] Scheiber AL Clark CA Kaito T Iwamoto M Horwitz EM Kawasawa YI . Culture condition of bone marrow stromal cells affects quantity and quality of the extracellular vesicles. Int J Mol Sci (2022) 23(3):1017. 10.3390/ijms23031017. 35162938 PMC8834965

[19] Cargnoni A Papait A Masserdotti A Pasotti A Stefani FR Silini AR . Extracellular vesicles from perinatal cells for anti-inflammatory therapy. Front Bioeng Biotechnol Front Media S.A (2021) 9doi: 10.3389/fbioe.2021.637737doi: . PMC7892960 33614619

[20] Magatti M Masserdotti A Bonassi Signoroni P Vertua E Stefani FR Silini AR . B lymphocytes as targets of the immunomodulatory properties of human amniotic mesenchymal stromal cells. Front Immunol (2020) 11doi: 10.3389/fimmu.2020.01156. PMC7295987 32582218

[21] Pianta S Bonassi Signoroni P Muradore I Rodrigues MF Rossi D Silini A . Amniotic membrane mesenchymal cells-derived factors skew T cell polarization toward treg and downregulate Th1 and Th17 cells subsets. Stem Cell Rev Rep (2015) 11(3):394–407doi: 10.1007/s12015-014-9558-4. 25348066 PMC4451472

[22] Magatti M de Munari S Vertua E Gibelli L Wengler GS Parolini O . Human amnion mesenchyme harbors cells with allogeneic T-cell suppression and stimulation capabilities. Stem Cells (2008) 26(1):182–92doi: 10.1634/stemcells.2007-0491. 17901399

[23] Pianta S Magatti M Vertua E Bonassi Signoroni P Muradore I Nuzzo AM . Amniotic mesenchymal cells from pre-eclamptic placentae maintain immunomodulatory features as healthy controls. J Cell Mol Med (2016) 20(1):157–69doi: 10.1111/jcmm.12715. PMC4717851 26515425

[24] Morales-Kastresana A Telford B Musich TA McKinnon K Clayborne C Braig Z . Labeling extracellular vesicles for nanoscale flow cytometry. Sci Rep (2017) 7(1):1878. doi: 10.1038/s41598-017-01731-2 28500324 PMC5431945

[25] Reddy M Eirikis E Davis C Davis HM Prabhakar U . Comparative analysis of lymphocyte activation marker expression and cytokine secretion profile in stimulated human peripheral blood mononuclear cell cultures: An *in vitro* model to monitor cellular immune function. J Immunol Methods (2004) 293(1–2):127–42doi: 10.1016/j.jim.2004.07.006. 15541283

[26] Beez CM Haag M Klein O van Linthout S Sittinger M Seifert M . Extracellular vesicles from regenerative human cardiac cells act as potent immune modulators by priming monocytes. J Nanobiotechnol (2019) 17(1):72. doi: 10.1186/s12951-019-0504-0 PMC6537224 31133024

[27] Khatri M Richardson LA Meulia T . Mesenchymal stem cell-derived extracellular vesicles attenuate influenza virus-induced acute lung injury in a pig model. Stem Cell Res Ther (2018) 9(1):17. doi: 10.1186/s13287-018-0774-8. 29378639 PMC5789598

[28] Bellio MA Kanashiro-Takeuchi RM Takeuchi L Kulandavelu S Lee YS Balkan W . Systemic delivery of large-scale manufactured wharton’s jelly mesenchymal stem cell-derived extracellular vesicles improves cardiac function after myocardial infarction. J Cardiovasc Aging (2022) 2:9. doi: 10.20517/jca.2021.21. 35112111 PMC8804674

[29] Zhao J Li X Hu J Chen F Qiao S Sun X . Mesenchymal stromal cell-derived exosomes attenuate myocardial ischaemia-reperfusion injury through miR-182-regulated macrophage polarization. Cardiovasc Res (2019) 115(7):1205–16. doi: 10.1093/cvr/cvz040 PMC6529919 30753344

[30] Riazifar M Mohammadi MR Pone EJ Yeri A Lasser C Segaliny AI . Stem cell-derived exosomes as nanotherapeutics for autoimmune and neurodegenerative disorders. ACS Nano (2019) 13(6):6670–88. doi: 10.1021/acsnano.9b01004 PMC6880946 31117376

[31] Roy A Mantay M Brannan C Griffiths S . Placental tissues as biomaterials in regenerative medicine. Tsai FM editor BioMed Res Int (2022) 2022:1–26. doi: 10.1155/2022/6751456 PMC9050314 35496035

[32] Pierce J Jacobson P Benedetti E Peterson E Phibbs J Preslar A . Collection and characterization of amniotic fluid from scheduled c-section deliveries. Cell Tissue Banking (2016) 17(3):413–25. doi: 10.1007/s10561-016-9572-7 27460879

[33] Weissenbacher T Laubender RP Witkin SS Gingelmaier A Schiessl B Kainer F . Influence of maternal age, gestational age and fetal gender on expression of immune mediators in amniotic fluid. BMC Res Notes (2012) 5:375. doi: 10.1186/1756-0500-5-375 22827842 PMC3479422

[34] Bishop EL Gudgeon N Dimeloe S . Control of T cell metabolism by cytokines and hormones. Front Immunol Front Media S.A (2021) 12:653605. doi: 10.3389/fimmu.2021.653605 PMC8076900 33927722

[35] Teng GG Wang WH Dai Y Wang SJ Chu YX Li J . Let-7b is involved in the inflammation and immune responses associated with helicobacter pylori infection by targeting toll-like receptor 4. PloS One (2013) 8(2):e56709. doi: 10.1371/journal.pone.0056709 23437218 PMC3577724

[36] Shigemoto-Kuroda T Oh JY Kim DK Jeong HJ Park SY Lee HJ . MSC-derived extracellular vesicles attenuate immune responses in two autoimmune murine models: Type 1 diabetes and uveoretinitis. Stem Cell Rep (2017) 8(5):1214–25. doi: 10.1016/j.stemcr.2017.04.008 PMC5425726 28494937

[37] Fan Y Herr F Vernochet A Mennesson B Oberlin E Durrbach A . Human fetal liver mesenchymal stem cell-derived exosomes impair natural killer cell function. Stem Cells Dev (2019) 28(1):44–55. doi: 10.1089/scd.2018.0015 30328799

[38] Del Fattore A Luciano R Pascucci L Goffredo BM Giorda E Scapaticci M . Immunoregulatory effects of mesenchymal stem cell-derived extracellular vesicles on T lymphocytes. Cell Transplant (2015) 24(12):2615–27. doi: 10.3727/096368915X687543 25695896

[39] Abu-Raya B Michalski C Sadarangani M Lavoie PM . Maternal immunological adaptation during normal pregnancy. Front Immunol Front Media S.A (2020) 11. doi: 10.3389/fimmu.2020.575197 PMC7579415 33133091

[40] Holtan SG Creedon DJ Haluska P Markovic SN . Cancer and pregnancy: Parallels in growth, invasion, and immune modulation and implications for cancer therapeutic agents. Mayo Clin Proc (2009) 84(11):985–1000. doi: 10.1016/S0025-6196(11)60669-1 PMC2770910 19880689

[41] Devvanshi H Kachhwaha R Manhswita A Bhatnagar S Kshetrapal P . Immunological changes in pregnancy and prospects of therapeutic pla-xosomes in adverse pregnancy outcomes. Front Pharmacol (2022) 13. doi: 10.3389/fphar.2022.895254 PMC9065684 35517798

[42] Watanabe M Iwatani Y Kaneda T Hidaka Y Mitsuda N Morimoto Y . Changes in T, b, and NK lymphocyte subsets during and after normal pregnancy. Am J Reprod Immunol (1997) 37(5):368–77. doi: 10.1111/j.1600-0897.1997.tb00246.x 9196795

[43] Fujisaki S Mori N Sasaki T Maeyama M . Cell-mediated immunity in human pregnancy changes in lymphocyte reactivity during pregnancy and postpartum. Microbiol Immunol (1979) 23(9):899–907. doi: 10.1111/j.1348-0421.1979.tb02823.x 317125

[44] Bai K Lee CL Liu X Li J Cao D Zhang L . Human placental exosomes induce maternal systemic immune tolerance by reprogramming circulating monocytes. J Nanobiotechnol (2022) 20(1):86. doi: 10.1186/s12951-022-01283-2 PMC8857816 35180876

[45] Mitrani MI Bellio MA Meglin A Khan A Xu X Haskell G . Treatment of a COVID-19 long hauler with an amniotic fluid-derived extracellular vesicle biologic. Respir Med Case Rep (2021) 34:101502. doi: 10.1016/j.rmcr.2021.101502 34485048 PMC8405236

[46] Donlan AN Sutherland TE Marie C Preissner S Bradley BT Carpenter RM . IL-13 is a driver of COVID-19 severity. JCI Insight (2021) 6(15):e150107. doi: 10.1172/jci.insight.150107 34185704 PMC8410056

[47] Phetsouphanh C Darley DR Wilson DB Howe A Munier CML Patel SK . Immunological dysfunction persists for 8 months following initial mild-to-moderate SARS-CoV-2 infection. Nat Immunol (2022) 23(2):210–6. doi: 10.1038/s41590-021-01113-x 35027728

